# The Association Between Resting State Functional Connectivity and the Trait of Impulsivity and Suicidal Ideation in Young Depressed Patients With Suicide Attempts

**DOI:** 10.3389/fpsyt.2021.567976

**Published:** 2021-07-28

**Authors:** Jun Cao, Xiaorong Chen, Jianmei Chen, Ming Ai, Yao Gan, Jinglan He, Li Kuang

**Affiliations:** ^1^Department of Psychiatry, The First Affiliated Hospital of Chongqing Medical University, Chongqing, China; ^2^Mental Health Center, University-Town Hospital of Chongqing Medical University, Chongqing, China

**Keywords:** youth suicide, depression, functional magnetic resonance imaging, impulsivity, resting-state functional connectivity

## Abstract

Suicide is a leading cause of death among youth and is strongly associated with major depressive disorder (MDD). However, the neurobiological underpinnings of suicidal behaviour and the identification of risk for suicide in young depressed patients are not yet well-understood. In this study, we used a seed-based correlation analysis to investigate the differences in resting-state functional connectivity (RSFC) in depressed youth with or without a history of suicide attempts and healthy controls (HCs). Suicidal attempters (ATT group, *n* = 35), non-suicide attempters (NAT group, *n* = 18), and HCs exhibited significantly different RSFC patterns with the left superior prefrontal gyrus (L-SFG) and left middle prefrontal gyrus (L-MFG) serving as the regions of interest (ROIs). The ATT group showed decreased RSFC of the left middle frontal gyrus with the left superior parietal gyrus compared to the NAT and HC groups. Decreased RSFC between the left superior frontal gyrus and the right anterior cingulate cortex (rACC) was found in the ATT group compared to the NAT and HC groups. Furthermore, the left prefrontal-parietal connectivity was associated with suicidal ideation and levels of impulsivity, but RSFC of the left prefrontal cortex with the rACC was correlated exclusively with impulsivity levels and was not related to suicidal ideation in the ATT group. Our results demonstrated that altered RSFC of the prefrontal-parietal and prefrontal-rACC regions was associated with suicide attempts in depressed youth, and state-related deficits in their interconnectivity may contribute to traits, such as cognitive impairments and impulsivity to facilitate suicidal acts. Our findings suggest that the neural correlates of suicidal behaviours might be dissociable from those related to the severity of current suicidal ideation. Neural circuits underlying suicide attempts differ from those that underlie suicidal ideation.

## Introduction

Suicide is an important public health problem worldwide (1) and a leading cause of death among youth aged 15–24 years in China (2). Youth suicide has elicited great concern from the public, policy-makers, and health care providers. In addition, suicide attempts occur 10–20 times more frequently than completed suicide and are known to be the most powerful predictor of future completed suicide (3). Thus, youth who survive a suicide attempt continue to be at risk for completed suicide (4). The majority of suicide victims or suicide attempters suffer from a mood disorder, the most frequent of which is major depressive disorder (MDD) (5). Improving our ability to predict and subsequently prevent suicidal behaviour in depressed patients has become an important priority. To date, the assessment of suicidal risk is based on sociodemographic and clinical factors, often yielding a high sensitivity but a low specificity (6). Evidence of a neurobiological basis of vulnerability to suicidal behaviour is increasing (7). Thus, a better understanding of the mechanisms underlying risk for a suicide attempt from a neuroscience perspective will provide a framework for conceptualising potential interventions. However, we have limited understanding of the neurobiological roots of suicidal behaviour, especially in adolescents. Because adolescence and early adulthood is a time of high risk for suicide as well as a period that is marked by significant neural development (8), investigating the neurobiological underpinnings of suicidality within this population is critical to better understand the psychopathology in its early stages and aid in developing intervention and prevention strategies that promote healthy neurodevelopmental trajectories.

Functional magnetic resonance imaging (fMRI) studies provide a promising strategy for learning about neurobiology that may confer risk for suicidal behaviour and potentially provide an objective neurobiological marker of suicidal risk in depressed youth (9). fMRI can help identify neurobiological underpinnings of pathophysiologic mechanisms that are not observable at the behavioural level and can also provide targets for future neurobiological interventions (9). Resting-state fMRI (RS-fMRI) has recently emerged as a useful tool for investigating brain functional connections without using externally controlled task paradigms. Resting-state functional connectivity (RSFC) analysis has been suggested as an established, powerful technique for an unbiased analysis that reveals correlations in the activity of discrete brain regions during rest (10), and it has been successfully employed to detect abnormal functional integration in several brain disorders and provide some important information for the understanding of these diseases, such as in Alzheimer's disease (11–13), heroin addiction (14), schizophrenia (15), and depression (16, 17). An increasing number of RSFC studies have investigated the associations between abnormal FC of particular brain regions and the pathophysiology of brain diseases (16, 18). The investigation of RSFC could provide important insights into the intrinsic FC in depressed youth with suicide attempts, and these studies will make important contributions to a better understanding of the neural circuitry underlying suicide in depression. However, the number of RSFC studies examining suicide in youth is small.

Previous neuroimaging studies have demonstrated the involvement of the prefrontal cortex (PFC) in suicidal behaviour in young adults and adolescents (9, 19–23). A model-based structural neuroimaging study with a translational perspective confirmed structural prefrontal alterations in adults with histories of suicide attempts (9). A near-infrared spectroscopy (NIRS) study revealed that MDD patients with suicidal ideation displayed reduced haemodynamic activation in prefrontal regions, and haemodynamic changes in the prefrontal cortex were negatively correlated with the severity of suicidal ideation in patients with MDD, suggesting that the PFC is a brain substrate of suicidal ideation in depressive states in adult patients with MDD (24). A previous study (25) found a relative hypometabolism in the middle frontal gyrus and superior parietal lobule in depressed adults with suicide plans when compared to depressed patients without suicidal ideation or suicide plans. Reduced left ventrolateral prefrontal cortex (VLPFC) volumes were found in attempters vs. non-suicide attempters and healthy controls aged between 18 and 60 (9, 22). Task-based fMRI studies have revealed that the activity of the right superior frontal gyrus is decreased in depressed adolescents with a history of suicide attempt in response to exposure to angry faces (26, 27), and adult males with a history of suicide attempt showed decreased activity in the right superior frontal gyrus in response to 100% intensity angry vs. neutral faces relative to healthy and depressed non-attempter controls (28). Another study found decreased bilateral perfusion in the superior/medial PFC in a cohort of depressed individuals who would later complete suicide (29). In adolescents, one study found that the dorsolateral PFC and temporoparietal junction was less activated with suicidal ideation than in same-age control subjects during passive viewing of negative stimuli (30). Our previous neuroimaging study on suicide in adolescents, we found decreased activity in the left superior prefrontal gyrus (L-SFG) and left middle prefrontal gyrus (L-MFG) in depressed patients with suicidal behaviour and decreased activity in the L-SFG and L-MFG was associated with increased impulsivity. Dysfunction of the L-SFG and L-MFG may underlie the behavioural disinhibition associated with impulsivity and increase the risk for suicide in depressed youth. In sum, these two brain areas seem to play an important role in the neurobiological mechanisms of suicidal behaviour and represent a potential neurobiological diathesis or predisposition to suicidal behaviour in depressed patients. These two brain areas might be suitable for functional neuroimaging studies on suicide.

In the current study, we selected the L-SFG and L-MFG as regions of interest (ROIs), and to investigate the association between RSFC and suicide attempts, we used a seed-based correlation analysis to explore and characterise abnormal resting-state FC patterns *in vivo* in depressed youth with a history of a suicide attempt relative to other depressed patients without a history of suicide attempt and healthy controls (HCs). We hypothesised that depressed youth who had a history of suicide attempt would show abnormal RSFC patterns of the L-SFG and L-MFG with other cortical regions, and there would be significant associations between aberrant RSFC and the traits of suicide-related behaviour.

## Method

### Participants

The Research Ethics Committee of the First Affiliated Hospital of Chongqing Medical University approved this study, and informed consent of all participants was obtained after the nature of the procedures had been fully explained. We would tell participants the purpose and specific content of this study, all participants would sign an informed consent before the study. We recruited 53 right-handed depressed youth from the Department of Psychiatry, the First Affiliated Hospital of Chongqing Medical University. The Structured Clinical Interview for Diagnostic and Statistical Manual IV (DSM-IV) Axis I disorders (SCID-I) (31) was administered to each patient by two qualified psychiatrists for the diagnosis of depressive disorder. The inclusion criteria were as follows: (a) unipolar subtype, (b) refrained from antidepressants for at least 1 month at the time of study and (c) total score of 17-item Hamilton Depression rating scale (HAMD) more than 17 and Beck Depression Inventory (BDI) 14 on the day of their scan. The exclusion criteria included any other psychotic disorders or comorbid DSM-IV axis I major psychiatric disorders, any history of significant head trauma with loss of consciousness, and other clinically relevant abnormalities in their medical history or laboratory examinations, and/or any contraindications for MRI scan. The patients were categorised into two groups: suicide attempters (ATT group, *n* = 35) with a history of at least one suicide attempt within the 6 months prior to magnetic resonance scanning and non-suicide attempters (NAT group, *n* = 18) without such a history. A suicide attempt was defined as self-destructive behaviour committed with some degree of intent to die and was assessed using the Columbia Suicide History Form, and the history of suicide attempts for a patient was confirmed based on the documentation of suicide attempts in their medical records or a history of visits to the hospital emergency department after attempting suicide. The severity of depression was assessed by using the HAMD and BDI, and the Scale for Suicide Ideation (SSI) was used to evaluate suicidal ideation severity. Additional assessments used were the Beck Hopelessness Scale (BHS) and the Barratt Impulsivity Scale, Version 11 (BIS-11).

Additionally, 47 right-handed HCs with no history of neurologic or psychiatric diseases were recruited from among the friends and spouses of the patients and were matched with the patients for age, gender, and education.

### MRI Acquisition

MRI scans were performed on a 3T GE Signa HDxt MRI system (General Electric Healthcare, Chicago, Illinois, USA) using a standard eight-channel head coil. RS-fMRI data were acquired using an echo-planar image (EPI) pulse sequence at 2-s intervals for a total of 8 min, employing the following imaging parameters: 33 axial slices, repetition time (TR) = 2000 ms, echo time (TE) = 40 ms, slice thickness = 4.0 mm, flip angle = 90°, field of view (FOV) = 240 × 240 mm^2^, and in-plane resolution = 64 × 64. A total number of 240 time points were axially recorded. Foam pads were used to minimise head motion, and the participants were instructed to relax in a comfortable position with their eyes closed, to remain still and awake. In addition, a high-resolution 3D T1-weighted MRI image to which the RS-fMRI data were to be coregistered was obtained using a fast gradient echo (FGRE) sequence, TR/TE = 24 ms/9 ms, flip angle = 90°, thickness/gap = 1.0/0 mm, FOV = 24 × 24cm, and matrix = 256 × 256. No obvious brain abnormalities on conventional MRI were observed, and none of the subjects felt discomfort during or after the procedure.

### Pre-processing of fMRI Data

The RS-fMRI data were pre-processed using Data Processing and Analysis for (Resting-State) Brain Imaging (DPABI) software (32) (http://www.restfmri.net) running in MATLAB (Mathworks, Natick, MA, USA). The pre-processing steps included slice timing correction, realignment for head motion, spatial normalisation in Montreal Neurological Institute (MNI) space, spatial smoothing, linear trend removal and filtering (0.01–0.08 Hz), and nuisance signal regression. The first 10 EPI time points were removed to ensure steady state, slice timing correction, realignment for head motion across the time series incorporating nuisance covariate regression using the Friston 24-parameter model (33) and use of signals from segmentations of the white matter (WM) and cerebrospinal fluid (CSF) compartments in the 3D T1-weighted image as regressors to reduce respiratory and cardiac effects. Additionally, linear trend removal and filtering (0.01–0.08 Hz) was included as a regressor to account for potential drifts in the BOLD signal. The resulting aligned image time series for each subject were each co-registered with the corresponding 3D T1-weighted image and the Diffeomorphic Anatomical Registration Through Exponentiated Lie Algebra (DARTEL) tool (34) was used to normalise the data for all subjects to Montreal Neurological Institute (MNI) space (resampling with 3 × 3 × 3 mm^3^ resolution), and spatially smoothed with a Gaussian kernel of 4 mm full-width half-maximum (FWHM).

Six parameters associated with head motion signals were regressed out (three for shift and three for rotation), and the overall head motion was calculated as the average magnitude of the head motion. No significant differences were found in the root mean square (RMS) head movement among the three groups (*p* = 0.35). All participants had <1 mm maximum displacement head movement in any direction (x, y, and z) and <1° maximum displacement in any angular dimension.

### Functional Connectivity Analysis

Based on previous literature and our prior study results, we selected the L-SFG and L-MFG as seed ROIs (9, 19–23), and the centred MNI coordinates of the seed regions were x, y, z = −33, 63, −6 (L-SFG) and x, y, z = −27, 30, 30 (L-MFG). The seed regions were defined by generating a 6-mm radius in the ROIs. A voxel-wise FC analysis was performed by computing the temporal correlation between the mean time series of each seed ROI and the time series of each voxel in the brain. The correlation coefficients (r) were then normalised to Z-scores with the Fisher r-to-z transformation using the following equation:

(1) $$\begin{array}{r} z=0.5\log\left[\frac{1+r}{1-r}\right] \end{array}$$

The Z-score maps of each ROI for all subjects were created, and they were defined as RSFC maps.

### Statistical Analysis

Demographic and clinical characteristics were analysed using IBM SPSS Statistics for Windows, Version 22.0 (Armonk, New York). For the FC analysis, one-sample *t*-tests were first conducted with the individual Z-maps for within-group comparisons (within the grey matter mask) in each group (*p* < 0.05), and a mask was created by combining the statistical maps of one-sample *t*-test results. Then, one-way analysis of variance (ANOVA) was used to compare the differences in RSFC among the three groups (ATT vs. NAT vs. HC) in the combined mask as above. Age, gender, education level, and mean framewise displacement (FD) were used as covariates. Statistical significance was set at corrected *p* < 0.05 (uncorrected *p* < 0.01) using Gaussian random field correction, which was performed using DPABI software. We further extracted Z values from the grey matter mask showing significant differences among the three groups, and *post hoc* two-sample *t*-tests of the Z values between each pair of groups (ATT vs. NAT, ATT vs. HC, NAT vs. HC) were applied to correct for multiple comparisons (*p* < 0.05, Bonferroni-corrected). Finally, two-tailed Pearson correlation analyses were separately performed to assess the relationships between RSFC and the HAMD, BDI, BIS-11, BHS, and SSI scores within the ATT group in SPSS 22.0. Statistical significance was determined following family-wise error (FWE) correction at *p* < 0.05.

## Results

### Demographics and Clinical Characteristics

**Figure 1:**
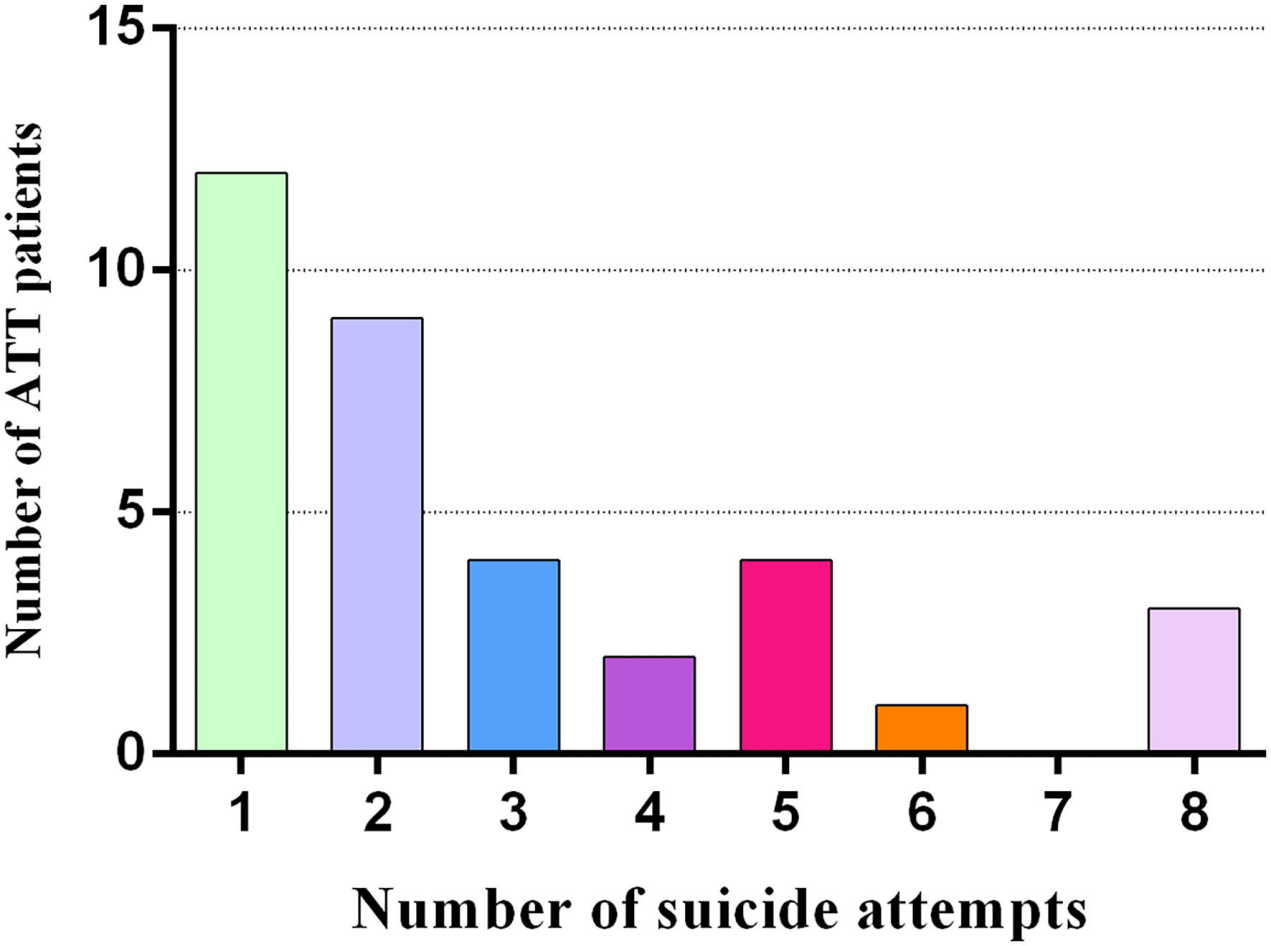
The number of suicide attempts in 35 ATT patients (who had attempted suicide 1 to 6 months prior to the fMRI scan).

Demographics and clinical characteristics are shown in Table 1. The patient groups (ATT and NAT group) had higher HAMD (20.03 ± 5.61 vs. 23.50 ± 8.92 vs. 3.85 ± 2.32, *p* < 0.001), BDI (23.91 ± 7.94 vs. 24.94 ± 7.14 vs. 3.94 ± 3.33, *p* < 0.001) and SSI scores (11.06 ± 4.71 vs. 10.22 ± 2.98 vs. 2.87 ± 2.31, *p* < 0.001) than the HC group. In addition, there were significant differences in impulsivity (BIS-11 total score) (73.86 ± 10.68 vs. 68.39 ± 8.81 vs. 61.79 ± 8.19, *p* < 0.001) and hopelessness (BHS total score) (12.14 ± 3.97 vs. 7.50 ± 2.41 vs. 3.79 ± 2.40, *p* < 0.001) among the three groups. By definition, the BIS-11 and BHS scores of the ATT patients were significantly higher than those of the NAT patients (*p* < 0.001). The number of suicide attempts in the ATT patients is displayed in Figure 1.

**Table 1:** Demographic and clinical characteristics of patients and healthy.

**Characteristic**	**ATT (*N* = 35)**	**NAT (*N* = 18)**	**HC (*N* = 47)**	***P*-value**	***post-hoc* test**
Age (years)	20.63 ± 3.65	21.26 ± 3.02	20.48 ± 1.86	0.584	-
Gender (male/female)	10/25	8/10	16/31	0.602	-
Education (years)	12.97 ± 1.90	13.22 ± 1.44	13.55 ± 1.52	0.289	-
BIS-11	73.86 ± 10.68	68.39 ± 8.81	61.79 ± 8.19	<0.001	ATT > NAT > HC
HAMD	20.03 ± 5.61	23.50 ± 8.92	3.85 ± 2.32	<0.001	ATT, NAT > HC
BDI	23.91 ± 7.94	24.94 ± 7.14	3.94 ± 3.33	<0.001	ATT, NAT > HC
BHS	12.14 ± 3.97	7.50 ± 2.41	3.79 ± 2.40	<0.001	ATT > NAT > HC
SSI	11.06 ± 4.71	10.22 ± 2.98	2.87 ± 2.31	<0.001	ATT, NAT > HC

### Group Differences in RSFC Analysis

**Figure 2:**
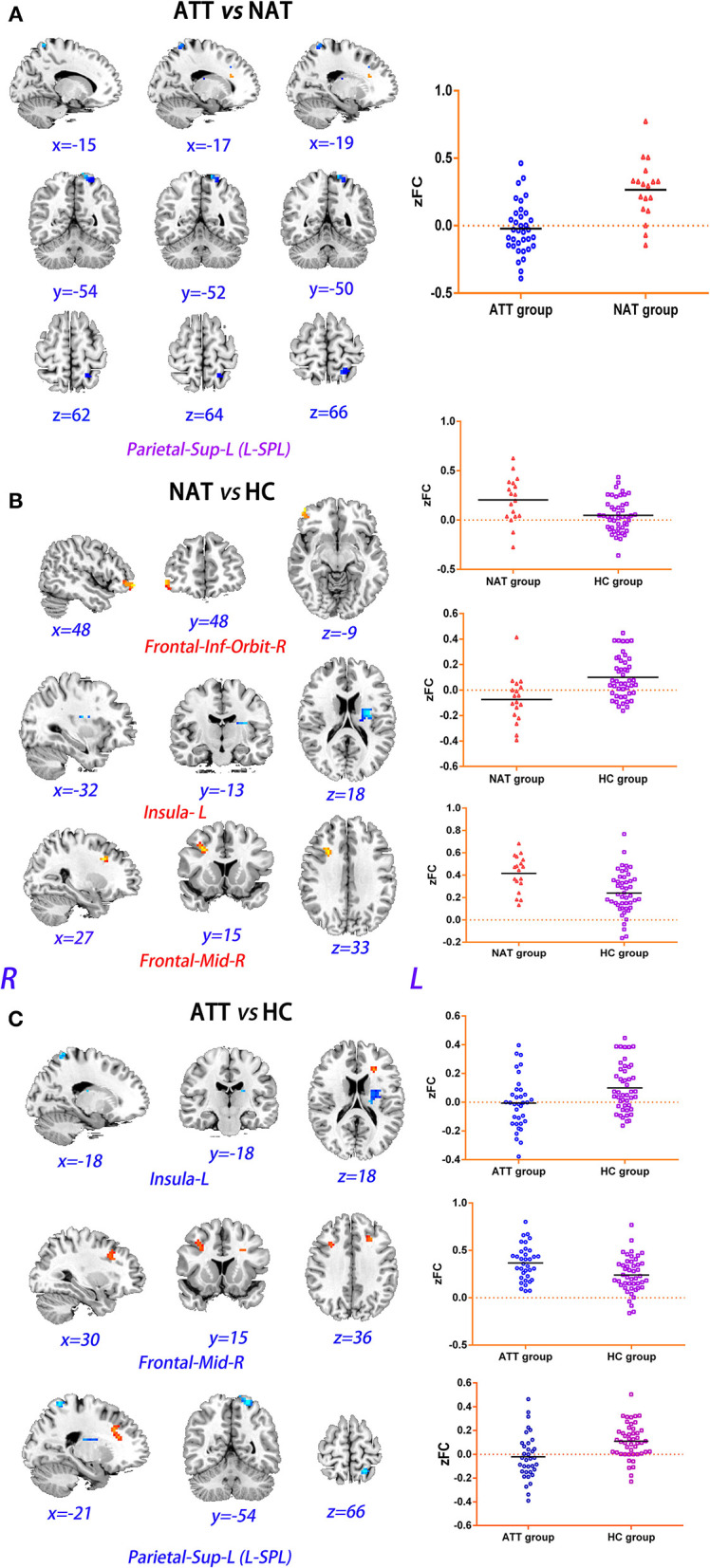
Group differences in RSFC analysis using L-MFG as seed of ROIs. **(A)** *post hoc t*-tests revealed that the ATT group demonstrated decreased RSFC between the L-MFG and L-SPG compared to the NAT group (FWE corrected, *p* < 0.05). **(B)** Compared to HC group, NAT group showed decreased RSFC of the L-MFG with the left insular cortex, as well as with the right inferior frontal grus and R-MFG (FWE corrected, *p* < 0.05). **(C)** Compared to the HC group, the ATT group showed decreased RSFC of the L-MFG with the left insular cortex and L-SPG, and increased RSFC between the L-MFG and R-MFG (FWE corrected, *p* < 0.05).

**Figure 3:**
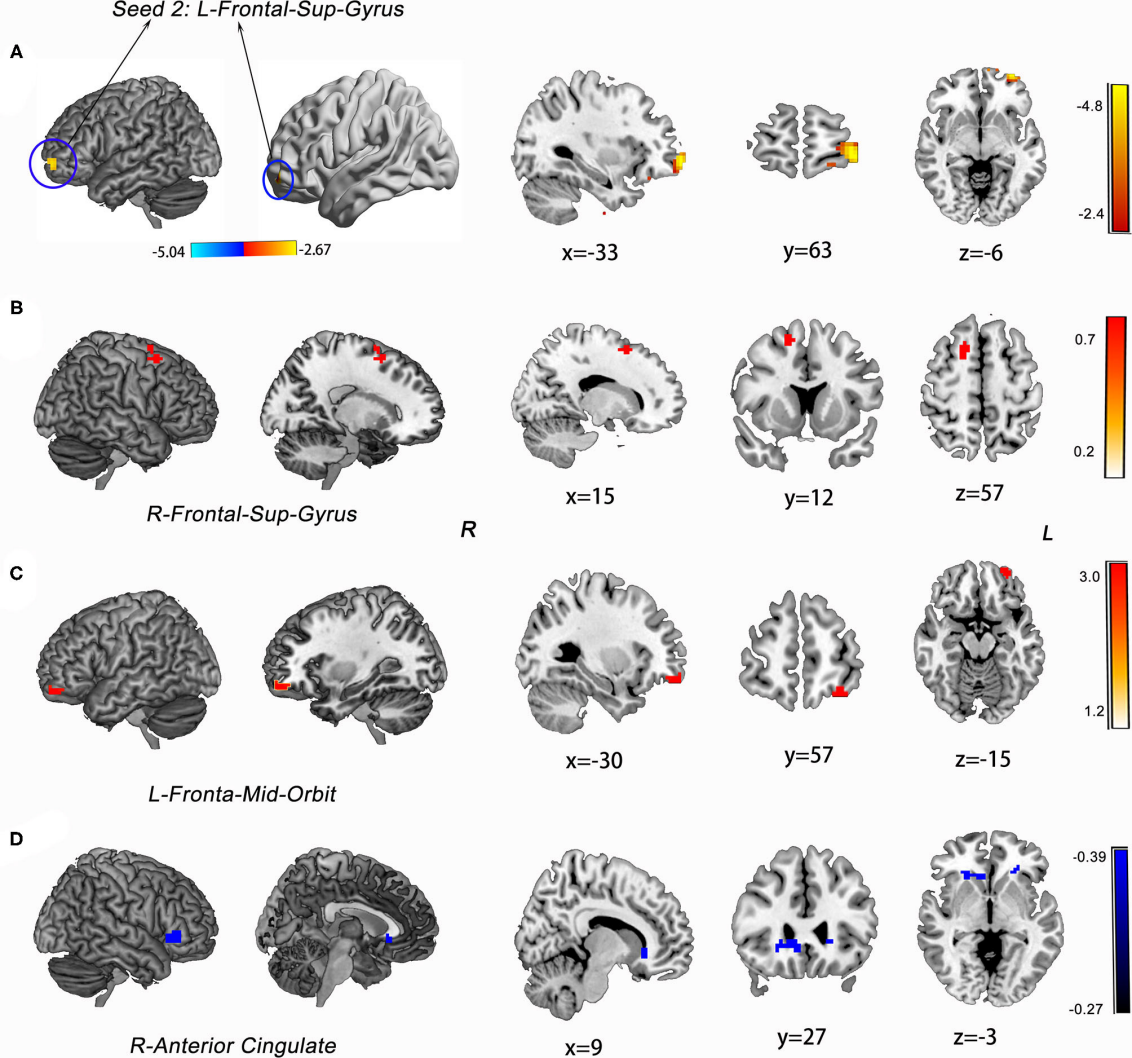
Group differences in RSFC analysis using L-SFG as seed of ROIs and the statistical maps showing *post hoc t-test* results of RSFC analysis among the three groups. **(A)** The map of seed region of interest (ROI): left superior frontal gyrus (L-SFG, Peak MNI: −33 63 −6). **(B)** ATT group vs. NAT group; **(C)** NAT group vs. HC group; **(D)** ATT group vs. HC group; Red and blue denote increased and decreased RSFC, respectively, and the colour bars indicate the *t*-value from *post-hoc* analysis between each pair of groups.

The ATT group demonstrated decreased RSFC between the L-MFG and L-SPG (FWE corrected, *p* < 0.05) compared to the NAT group. And decreased RSFC of the L-SFG with rACC (FWE corrected, *p* < 0.05) was found in the ATT group relative to both the NAT and HC groups. In addition, the NAT group showed decreased RSFC of the L-MFG with the left insular cortex, as well as with the L-SPG relative to the HC group (Table 2 and Figures 2, 3).

**Table 2:** Differences in left MFG and SFG seed functional connectivity among the ATT group, NAT group, and HC group.

**Brain area**	**MNI coordinates**			***T*-value**	**Voxels size**
	**x**	**Y**	**z**		
**Seed 1: left middle frontal gyrus (L-MFG, Peak MNI:** **−27 30 30)**					
**ATT** **<** **NAT**
Parietal-Sup-L	−17	−52	71	−1.22	4
**NAT** **>** **HC**
Frontal-inf-Orbit-R	48	48	−9	2.95	33
Frontal-Mid-R	27	15	33	3.46	35
**NAT** **<** **HC**
Insula-L	−32	−13	18	−3.70	28
Parietal-Sup-L	−24	−54	66	−3.12	18
**ATT** **>** **HC**
Frontal-Mid-R	30	15	36	3.05	30
**ATT** **<** **HC**
Insula-L	−18	−18	18	−3.12	23
Parietal-Sup-L	−21	−54	66	−3.64	33
**Seed 2: left superior frontal gyrus (L-SFG, Peak MNI:** –**33 63** –**6)**					
**ATT** **>** **NAT**
Frontal-Sup-R	15	12	57	3.96	24
**ATT** **<** **NAT**
Anterior Cingulate-R	24	30	−6	−2.46	32
**NAT** **>** **HC**
Frontal-Mid-Orbit-L	−30	57	−15	4.34	22
**ATT** **<** **HC**
Anterior Cingulate-R	9	27	−3	−4.31	32

### Correlations Between RSFC and Clinical Characteristics

**Figure 4:**
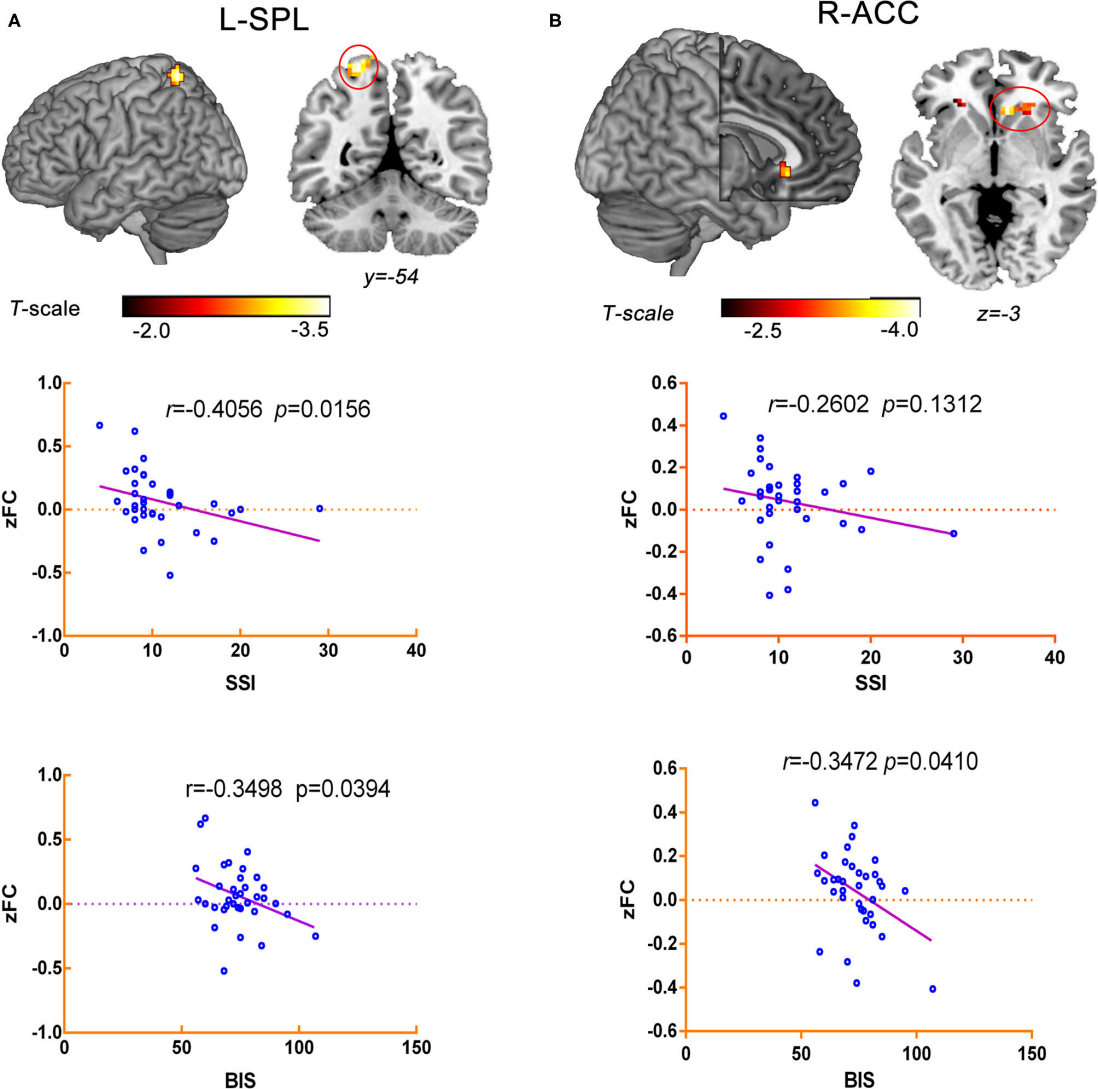
Correlation analysis between aberrant RSFC and the traits of suicide-related behaviour in ATT group. **(A)** The RSFC between the L-MFG and L-SPG was negatively associated with SSI scores (*r* = −0.4056, *p* = 0.0156) and BIS scores (*r* = −0.3498, *p* = 0.0394). **(B)** The RSFC of the L-SFG with the rACC was negatively correlated with BIS-11 scores (*r* = −0.3472, *p* = 0.0410), but it was not associated with the SSI total scores (*r* = −0.2602, *p* = 0.1312).

The correlation analysis indicated that decreased RSFC between the L-MFG and L-SPG was negatively associated with SSI scores (*r* = −0.4056, *p* = 0.0156) and BIS scores (*r* = −0.3498, *p* = 0.0394) in the ATT group. Additionally, the RSFC of the L-SFG with the rACC was negatively correlated with BIS-11 scores (*r* = −0.3472, *p* = 0.0410) within the ATT group, but no significant negative correlation of the SSI total scores with the RSFC between the L-SFG and rACC was observed in this group (*r* = −0.2602, *p* = 0.1312) (Figure 4).

## Discussion

In the current study, we investigated the differences in RSFC in depressed youth who had (suicide attempters; ATT group) or did not have a history of suicide attempts (NAT group) and healthy controls (HC). Decreased RSFC of the left middle frontal gyrus with the left superior parietal gyrus and decreased RSFC between the left superior frontal gyrus and the right anterior cingulate cortex (rACC) was found in the ATT group compared to the NAT and HC groups. Furthermore, the left prefrontal-parietal connectivity was associated with suicidal ideation and levels of impulsivity, but RSFC of the left prefrontal cortex with the rACC was correlated only with impulsivity levels and was not related to suicidal ideation in the ATT group. These results indicated that the decreased RSFC of the left PFC with the left parietal cortical areas and with the rACC may be implicated in the neurobiology of suicidal behaviour in young depressed patients. The findings suggest that impaired neural circuit connections may be vulnerability factors and the manifestations of suicide. This study would contribute to the detection of suicide risk and provide insight in a neurobiological target for suicide interventions in youth depression at the clinical level.

### Prefrontal-Parietal RSFC

Abnormal RSFC of the left PFC with left parietal cortical areas was observed in young depressed patients with suicide attempts in this study. The left hemisphere exhibited particular features with regards to not only general emotion processing but also specific depression pathophysiology (35), and a greater left hemispheric response to positive stimuli was noted during general emotion processing (36).

Elsewhere, in our study, we found the RSFC of the fronto-parietal circuit was correlated with the SSI and BIS-11 scores in the ATT group. The results of our study provide preliminary evidence that an abnormal left fronto-parietal circuit may be the underlying neural circuit related to suicidal behaviour associated with depression. The reduced resting-state metabolic activity in frontopolar and parietal brain regions that are involved in decision-making and choice, more particularly in exploratory behaviour, was associated with suicide plans in depressed individuals. Furthermore, another RSFC study found that MDD patients with suicidal ideation (SI group) exhibited decreased intrinsic functional connectivity (iFC) between the orbitomedial prefrontal cortex (OMPFC) and the rACC (37).

Elevated trait impulsivity in suicidal depressed patients was observed in our study, which was reported in a previous study. A preponderance of evidence has suggested that impulsivity (as measured by the BIS-11) likely predisposes individuals to suicide. The middle frontal gyrus is considered to play a role in reorienting attention, where both ventral and dorsal attention networks converge. Abnormal functioning and connectivity within the middle frontal gyrus have been documented in a meta-analysis as well as in a more recent study of adults with MDD (38). Furthermore, reduced cortical thickness of the middle frontal gyrus has been related to impulsivity in adolescents (39) and has been associated with motor impulsivity in particular (40). The neural substrate underlying impulsivity in suicidal depressed patients has also been illustrated in our previous research, which demonstrated that decreased activity in the L-MFG was associated with increased impulsivity in young suicidal depressed patients. In the present study, we found that decreased prefrontal-parietal RSFC was negatively correlated with elevated BIS-11 impulsivity scores in the ATT group. The correlation with BIS-11 scores demonstrated that impulsivity and prefrontal-parietal connectivity is highly relevant to our preliminary finding, further supporting the role of the PFC in suicide. In addition, adolescents with MDD and high suicidality showed lower activation in the medial PFC and anterior cingulate cortex (ACC) compared to MDD adolescents with low suicidality and HCs (41). Some research (41, 42) proposed that vulnerability to suicidal behaviour can be attributed to increased self-focus and hopelessness derived from emotional dysregulation in combination with abnormalities in the fronto-limbic or fronto-parietal-cerebellar pathways. These findings suggest that the altered fronto-parietal connectivity implicated in affective and cognitive processing procedures might contribute to the pathogenesis of suicidal behaviour in MDD.

The fronto-parietal circuit is also involved in goal-directed top-down processing, attention and executive function, decision-making and conflict resolution and is engaged in cognitive control (43). The aberrant FC of the prefrontal region with the parietal cortex may impair decision-making and cognitive control, which increases suicidal vulnerability and suicidal risk. Thus, the disruption of the fronto-parietal circuit may be involved in the pathogenesis of suicidal behaviour in depressed youth, and it may be implied as underlying neural substrate. However, it would necessitate further research to replicate and verify this finding.

### Prefrontal-rACC RSFC

In addition to the decreased connectivity in prefrontal-parietal circuits, reduced connectivity between L-SFG and rACC was also observed in the suicidal depressed patients compared to non-suicidal depressed patients. Our results are in agreement with several results from previous studies in depressed patients with and without suicide behaviour (25, 30, 44). A comparative study of regional cerebral glucose metabolism (rCMRglu) using 18-FDG-PET demonstrated that compared to depressed patients without suicidal ideation or suicide plans, depressed patients with suicidal ideation and suicide plans showed hypometabolism in the L-SFG, and comparing depressed patients with suicidal ideation to those without suicidal ideation revealed an association between suicidal ideation and decreased metabolism in the right cingulate gyrus (25, 30). One study (44) found that youths with suicide attempts exhibited increased activation in the right anterior cingulate gyrus and left dorsolateral PFC (dlPFC) while viewing negative facial expressions during an emotion perception task. Compared to those without suicidal ideation and HCs, depressed patients with suicidal ideation showed decreased RSFC between the rostral ACC and the middle temporal pole (37). Schreiner et al. (45) reported that higher suicidality was associated with lower RSFC between the posterior cingulate cortex (PCC) and a cluster encompassing the left superior and middle frontal gyri and superior parietal lobule. In addition, one study showed adolescents with MDD and suicide attempts had reduced FC between the ACC and bilateral insula when viewing angry faces (44), suggesting further aberrations in affective experience. Taken together, these findings indicate that functional abnormalities in the associations between the PFC and ACC are related to a vulnerability to suicide attempts.

As has been highlighted in the literature, reduced connectivity of the dorsal anterior cingulate cortex (dACC) with the medial thalamus and left pallidostriatum was found in patients suffering from depression, and there was a trend for decreased connectivity between the ACC and the amygdala (46). The orbito-frontal cortex (OFC), which receives connections from the amygdala and thalamus, has a significant role in the interpretation of stimuli in the environment, notably in attributing value to stimuli (stimuli–outcome association), which may be important for the triggering of the suicidal crisis in the face of environmental stressors. The lateral PFC receives motivational inputs from ACC and represents cognitive information from memory, which is deficient in suicide attempters. Dysfunction of this interconnected prefrontal network may, therefore, be instrumental in the suicidal process by corrupting information acquisition and processing, resulting in impaired decision-making. It would be reflected by negative assessments of life events and the automatic triggering of intense emotional responses, and the inability to control the evoked emotional responses and particular negative thoughts (including hopelessness, ruminations, and suicidal ideas), and to prevent choosing to commit a suicidal act over alternative options.

In the present study, the BIS-11 score was significantly associated with RSFC values between the L-SFG and rACC in the suicide attempt group, but there was no significant association between L-SFG-rACC connectivity and SSI scores. It is possible that the neural circuits that underlie suicidal behaviour are different from those that underlie suicidal ideation. Furthermore, our findings suggested that abnormal RSFC between the L-SFG and rACC may not be associated with the severity of current levels of suicidal ideation but might be related to a trait of suicidal behaviour. This study also provided evidence that the neural correlates of suicidal behaviours might be dissociable from those related to the severity of current suicidal ideation (47, 48). Therefore, we speculated that it will be necessary to identify traits and biological neuroimaging markers which are related to suicidal behaviour rather than suicidal ideation in early prevention and intervention of suicide.

The superior frontal gyrus and ACC play a key role in affect regulation, self-referential processing and inhibition of responding, and impulsivity has been more specifically associated with the inability to inhibit responding (49). A previous study revealed that reduced cortical thickness of the middle and superior frontal gyrus was associated with impulsivity in adolescents (39) and was linked to motor impulsivity in particular (40). The literature to date has suggested that suicidal patients have more difficulty inhibiting responses, resulting in more commission errors than non-suicidal patients. Therefore, abnormal prefrontal-rACC RSFC might prompt aberrant affect regulation and response inhibition, which results in high levels of impulsivity that facilitate suicidal acts.

### Limitations

Several limitations of our study should be acknowledged. First, this was a case–control design study, and suicidal behaviour was retrospectively assessed by psychiatrists and medical records. Longitudinal prospective studies are needed to follow young depressed patients to compare alterations in RSFC before and after a suicide attempt and track the changes in FC. Second, we did not evaluate the effects of antidepressant medications taken by the young depressed patients on the findings. The effects of medications or other treatments on functional connectivity were not evaluated and therefore, represent a confounding factor. Thus, pre-enrolment medications should be controlled in future studies investigating the altered physiology of the brain in patients with suicidal depression. Future research we will recruit first-episode drug-naive depressed patients to exclude the influence of pre-enrollment medications or other treatments on the results. Finally, we did not perform multiple comparison corrections when exploring the relationships between RSFC values and clinical scales, and therefore replication of these findings is required. In addition to the above limitations, the relatively modest group size may have influenced the statistical power, and future studies are needed to validate the findings in a larger sample size.

### Conclusion

In summary, we found hypoconnectivity of the L-MFG with the L-SPG and the L-SFG with the rACC in the ATT group compared with NAT group. In addition, the left prefrontal-parietal connectivity was associated with SSI and BIS-11 scores. In contrast, the RSFC of the left PFC with the rACC was correlated only with BIS-11 scores and was not related to SSI scores. Our findings may suggest that the neural correlates of suicidal behaviours might be dissociable from those related to the severity of current suicidal ideation. There may be different neural circuits underlying suicide attempts from those that underlie suicidal ideation. The state-related deficits in the RSFC of prefrontal areas with the parietal cortex and rACC may contribute to traits such as cognitive impairments or impulsivity to facilitate suicidal acts. These results may contribute to the development of neurobiologically informed interventions that are targeted towards normalising aberrant connectivity related to suicidality in young depressed patients in the future.

## Data Availability Statement

The raw data supporting the conclusions of this article will be made available by the authors, without undue reservation.

## Ethics Statement

The studies involving human participants were reviewed and approved by The Research Ethics Committee of the First Affiliated Hospital of Chongqing Medical University. Written informed consent to participate in this study was provided by the participants' legal guardian/next of kin. Written informed consent was obtained from the individual(s), and minor(s)' legal guardian/next of kin, for the publication of any potentially identifiable images or data included in this article.

## Author Contributions

JC and XC designed the trial and analysed the data. YG and JH recruited and scheduled participants and collected the fMRI data. JC and MA tested the participants. JC wrote the report. LK designed the study and revised the manuscript. All authors have read and approved the submission of this manuscript.

## Conflict of Interest

The authors declare that the research was conducted in the absence of any commercial or financial relationships that could be construed as a potential conflict of interest.

## Publisher's Note

All claims expressed in this article are solely those of the authors and do not necessarily represent those of their affiliated organizations, or those of the publisher, the editors and the reviewers. Any product that may be evaluated in this article, or claim that may be made by its manufacturer, is not guaranteed or endorsed by the publisher.
